# Plasma neutrophil extracellular traps in patients with sepsis-induced acute kidney injury serve as a new biomarker to predict 28-day survival outcomes of disease

**DOI:** 10.3389/fmed.2024.1496966

**Published:** 2024-11-19

**Authors:** Jian He, Feng Zheng, Lihua Qiu, Yilan Wang, Jing Zhang, Hongwei Ye, Qian Zhang

**Affiliations:** Department of Intensive Care Unit, First People’s Hospital of Changshu City, Changshu Hospital Affiliated to Soochow University, Changshu, Jiangsu, China

**Keywords:** acute kidney injury, inflammation, neutrophil extracellular traps, sepsis, survival

## Abstract

**Background:**

There is currently no accurate, readily available, or validated biomarker for assessing disease severity and survival outcomes in sepsis-induced acute kidney injury (SAKI), which limits the ability to conduct effective therapeutic interventions. The neutrophil extracellular traps (NETs) may be involved in the pathophysiology of SAKI. The present study investigated the predictive value of plasma NETs for the survival outcome of patients with SAKI.

**Methods:**

This observational study included 136 SAKI patients, all of whom underwent a 28-day follow-up. According to the follow-up records, SAKI patients were divided into two groups: the non-survivor group (60 subjects) and the survivor group (76 subjects). Blood samples were collected after the diagnosis of AKI to measure three NET markers and 12 inflammatory indices. Correlation analysis, logistic regression analysis, receiver operating characteristic curve analysis, and survival analysis were performed.

**Results:**

Compared to survivors, non-survivors among SAKI patients exhibited significantly higher levels of three plasma NET markers (all *p* < 0.001). Meanwhile, in SAKI patients, plasma levels of NET markers were significantly associated with serum levels of inflammatory indices. Additionally, serum interleukin (IL)-2, IL-8, IL-10, and tumor necrosis factor-alpha showed an interactive effect with plasma NET markers on the survival of SAKI patients. Furthermore, the combination of three plasma NET markers could identify SAKI patients with a poor 28-day survival with better accuracy (area under the curve **=** 0.857). Finally, plasma NET markers may independently predict the 28-day survival in SAKI patients.

**Conclusion:**

Plasma NET markers were elevated in SAKI patients with poor outcomes and served as biomarkers for predicting 28-day survival in SAKI patients.

## Introduction

Sepsis is a prevalent and highly frequent severe infection, primarily characterized by systemic inflammatory reactions (1–3). In clinical practice, sepsis can cause the occurrence of multiple organ failure, and sepsis-induced acute kidney injury (SAKI) shows an overall incidence of 40–60% (4). Previous studies have indicated that SAKI patients often have a poor prognosis in the intensive care unit (ICU) and experience significantly higher hospital mortality rates (5). Although published studies indicated that early continuous renal replacement therapy (CRRT) in SAKI could remove middle and small molecular solutes through convection and eliminate inflammatory mediators through adsorption (6), some findings also revealed that early CRRT could induce uncontrollable risks for patients, including thrombosis from vascular access, infection, and hemorrhage (7–10). Furthermore, previous studies have demonstrated that the mortality of SAKI patients is associated with urine output on the first day, serum creatinine (sCr), and platelet count (11–13), suggesting that these indicators may be useful predictors of mortality in SAKI patients. However, biomarkers predicting the survival of SAKI have not yet been determined. Hence, the current scarcity of effective and easily accessible biomarkers that can indicate disease severity and clinical outcome in SAKI poses a significant hindrance to investigating its pathogenesis and implementing therapeutic interventions.

The neutrophil extracellular traps (NETs) are extracellular, web-like structures composed of double-stranded DNA, histone, and granule proteins (14, 15). The generation of reactive oxygen species and the activation of nuclear peptidyl arginine deiminase-4 (PAD4) are involved in the formation and release of NETs (16, 17). Citrullination of histone H3 (citH3), neutrophil elastase-DNA (NE-DNA), and myeloperoxidase-DNA (MPO-DNA) are the common makers to represent the expression levels of NETs (14). A recent study observed that the increase in NETs in the glomeruli and peritubular capillaries of AKI mice models was associated with the severity of AKI and reducing NET levels could improve the renal pathological changes and creatinine levels (18). PAD4-deficient animal models could not form NETs and were protected from renal ischemia−/reperfusion-induced AKI (19). In patients with COVID-19-associated AKI, the excessive formation of NETs can swiftly lead to vessel occlusion and disrupt microcirculation, ultimately resulting in kidney dysfunction (20). Furthermore, in cardiac patients undergoing surgery with cardiopulmonary bypass, elevated NET levels causing endothelial damage serve as the most accurate predictor for the early onset of AKI (21). Furthermore, sepsis research has indicated that NETs may cause damage to endothelial cells and exacerbate the inflammatory response, leading to life-threatening organ failure (15, 22, 23). However, the investigation of NETs in SAKI is still in its early stages, and the potential role of NETs in predicting the prognosis of SAKI patients remains unclear.

The present study aimed to investigate changes in plasma NET markers during the early stage of SAKI and evaluated their predictive value for the 28-day survival outcome of SAKI patients. Additionally, we concurrently detected the expression of inflammation factors to evaluate the interaction between NETs and inflammation factors on the survival of SAKI patients.

## Materials and methods

### Participants and study design

All participants or their legal guardians provided written informed consent, and the Ethics Committee of the Changshu Hospital Affiliated to Soochow University approved the present study (Approval number: X202250).

This study included 136 SAKI patients, who were assessed between January 2021 and December 2023. All patients were obtained from Changshu Hospital Affiliated to Soochow University (Suzhou, China). According to the guidance from the KDIGO Clinical Practice Guidelines for Acute Kidney Injury and Acute Kidney Injury Network (AKIN) classification (24, 25), all patients were diagnosed and graded. Patients with an increase in serum creatinine (sCr) ≥ 26.5 μmol/L within 48 h or 1.5 times the baseline sCr value within 7 days were defined as AKI. In the present study, all patients were classified as stage 2 or stage 3. Meanwhile, the recognition of sepsis was performed according to the last revised definition of sepsis (26).

The exclusion criteria were as follows: (1) age less than 18 years; (2) receipt of nephrotoxin within 4 weeks prior to the study; (3) death within 72 h of hospital admission; (4) severe cardiovascular and cerebrovascular diseases, malnutrition, trauma, immunodeficiency, coagulation disorders, severe hepatitis, or tumors; (5) psychiatric disorders or drug addiction; (6) pregnancy; (7) poor adherence; and (8) non-infectious AKI without meeting the criteria for sepsis.

A follow-up was performed for each patient after diagnosis for 28 days or until death. Furthermore, according to the survival outcome of follow-up, the patients were further divided into two groups: the survivor group and the non-survivor group.

### Clinical data collection

The clinical data including demographic characteristics, infection sites, and combined diseases, were collected from patients’ electronic medical records. Meanwhile, laboratory examinations, including sCr, blood urea nitrogen (BUN), and estimated glomerular filtration rate (eGFR), of each patient were also collected from their first electronic medical records. Furthermore, the Acute Physiology and Chronic Health Evaluation II (APACHE II) score and the Sequential Organ Failure Assessment (SOFA) score were assessed after the diagnosis of AKI.

### Blood sample collection and detection

After the diagnosis of SAKI, peripheral venous blood was collected from each individual using a coagulation tube and an EDTA-coated tube between 6:00 am and 7:00 a.m. Within 30 min of collection, the samples of the coagulation tube were centrifuged at 3,500 rpm at 4°C for 10 min, and the serum was obtained and stored at −80°C until further use. Meanwhile, the plasma samples were obtained by centrifugation at 2,000 *g* at 4°C for 10 min and were stored at −80°C until further use.

The plasma levels of three circulating NET markers, citrullination of citH3, NE-DNA, and MPO-DNA, were detected after the diagnosis of AKI, using commercial enzyme-linked immunosorbent assays kit (citH3: HB1518-Hu; NE-DNA: HB3168-Hu; MPO-DNA: HB2223-Hu; Hengyuan Biotech Co., Ltd., Shanghai, China). The levels of these indicators were measured in triplicate, and the inter- and intra-assay coefficients of variation were < 5%.

The Luminex xMAP array procedure was performed according to the manufacturer’s instructions to measure the serum levels of inflammation indicators. The human 12-plex panel (Product ID: FCSTM09-12) was used based on the Luminex 200 multiplexing platform (Luminex Corp., Texas, United States) (27–29) after the diagnosis of AKI. For all the biological specimens, each sample was tested in triplicate, and their average concentrations were calculated for further statistical analysis.

### Sample size estimation

The sample size calculation was conducted using an online sample size calculator.^1^ The sample size used in the present study was acceptable based on the results of sample size calculation (*α* = 0.05, *β* = 0.2).

### Statistical analyses

Statistical analyses were performed using SPSS version 22.0 (SPSS Inc. Chicago, IL, United States) and R software (version 4.2.1). Continuous variables were presented as the mean ± standard deviation. The Kolmogorov–Smirnov test was utilized to identify the normal distribution of the data. An independent-sample *t*-test was used if variables were normally distributed for continuous variables. Categorical variables were analyzed using the chi-square test. The correlation analysis was performed using Pearson’s correlation coefficients, and logistic regression was used to evaluate the interaction between NETs and inflammation indices on the severity of SAKI. Receiver operating characteristic (ROC) curves were used to assess the accuracy of the markers in identifying non-survivors. Binary logistic regression was performed to compute a predicted value of each patient for obtaining a combined indicator based on NET markers (30). The Youden index (31) was utilized to determine the optimal value of sensitivity and specificity. Furthermore, to evaluate the prognostic significance of the NET markers for the 28-day survival, the Cox regression analysis was performed and the Kaplan–Meier curves were plotted. *p* < 0.05 were considered to indicate statistically significant differences.

## Results

### Clinical characteristics and laboratory examinations of participants

According to the records of follow-up, 76 SAKI patients were included in the survivor group, and the other 60 patients were included in the non-survivor group (Table 1).

**Table 1:** Clinical characteristics and laboratory examination of participants of two groups.

	Survivor (*N* = 76)	Non-survivor (*N* = 60)	*P*-value
Sex, male	44 (57.89%)	32 (53.33%)	0.707^b^
Age, years	53.21 ± 13.42	57.47 ± 18.10	0.269^a^
BMI	23.19 ± 3.82	23.63 ± 3.29	0.614^a^
Infection sites			0.491^b^
Pulmonary	34 (44.74%)	28 (46.67%)	–
Urinary tract	24 (31.58%)	12 (20.00%)	–
Biliary tract	18 (23.68%)	20 (33.33%)	–
Combined disease			
Type 2 diabetes	20 (26.32%)	22 (36.67%)	0.359^b^
Hypertension	22 (28.95%)	30 (50.00%)	0.076^b^
Coronary heart disease	20 (26.32%)	22 (36.67%)	0.359^b^
CKD	20 (26.32%)	19 (31.77%)	0.493^b^
sCr (μmol/L)	344.29 ± 23.04	350.97 ± 24.74	0.255^a^
eGFR (mL/min per 1.73 m^2^)	32.47 ± 4.65	32.67 ± 5.11	0.871^a^
BUN (mg/dL)	41.01 ± 6.56	44.02 ± 8.80	0.125^a^
APACHE II score	25.00 ± 3.80	25.87 ± 4.03	0.367^a^
SOFA score	9.00 ± 1.66	8.43 ± 2.08	0.216^a^
Treatment			
CRRT	54 (71.05%)	41 (68.33%)	0.732^b^
Vasoactive agents	28 (36.84%)	29 (48.33%)	0.177^b^
Glucocorticoids	23 (30.26%)	21 (35.00%)	0.558^b^

Data are presented as means (standard deviation) or number of participants in each group (% of total). BMI, body mass index; CKD, chronic kidney disease; sCr, serum creatinine; BUN, blood urea nitrogen; eGFR, estimated glomerular filtration rate; APACHE II, Acute Physiology and Chronic Health Evaluation II; SOFA, Sequential Organ Failure Assessment; CRRT, continuous renal replacement therapy.

a Independent-samples *t*-test.

b Chi-squared test.

As shown in Table 1, there were no significant differences in baseline clinical features, including age, gender, BMI, infection sites, combined diseases, smoking, and drinking habits, between the survivor and non-survivor groups (all *p* > 0.05). Furthermore, no significant differences were observed between the survivors and non-survivors in terms of the serum levels of sCr, BUN, and eGFR (Table 1, all *p* > 0.05). Meanwhile, the scores of APACHE II and SOFA assessments showed no significant difference between the two groups (Table 1, all p > 0.05).

### Detection of inflammatory indices and NETs

Patients in the non-survivor group have significantly higher plasma levels of NETs (citH3, NE-DNA, and MPO-DNA) than those in the survivor group (Table 2, all *p* < 0.05). Furthermore, a subgroup analysis further indicated that there was no significant difference in the plasma NET levels between patients with CKD and patients without CKD in both the survivor and non-survivor groups; however, survivors with or without CKD showed significantly increased NET levels as compared to non-survivors with or without CKD (Supplementary Figure S1).

**Table 2:** Serum levels of 13 inflammatory indices and plasma levels of NETs in participants of two groups.

	Survivor (*N* = 76)	Non-survivor (*N* = 60)	*P*-value
GM-CSF (ng/mL)	687.89 ± 91.31	738.47 ± 72.73	0.016
IFN-γ (pg/mL)	0.40 ± 0.08	0.43 ± 0.11	0.224
IL-1β (pg/mL)	130.43 ± 12.16	139.88 ± 15.88	0.007
IL-2 (pg/L)	1.39 ± 0.58	1.71 ± 0.65	0.003
IL-4 (pg/mL)	2.34 ± 0.24	2.50 ± 0.31	0.017
IL-5 (pg/mL)	0.34 ± 0.21	0.38 ± 0.17	0.407
IL-6 (pg/mL)	79.26 ± 7.72	85.40 ± 9.79	0.005
VEGF (pg/mL)	60.69 ± 8.33	60.74 ± 8.14	0.979
IL-8 (pg/mL)	119.72 ± 18.14	137.11 ± 24.62	0.001
IL-10 (pg/mL)	4.40 ± 1.54	6.17 ± 2.05	<0.001
IL-12 (mg/L)	160.93 ± 25.87	157.43 ± 29.80	0.606
TNF-α (pg/mL)	2.12 ± 0.29	2.38 ± 0.25	<0.001
citH3 (pg/mL)	256.84 ± 21.93	278.93 ± 23.82	<0.001
NE-DNA (ng/L)	231.73 ± 31.79	257.22 ± 22.64	<0.001
MPO-DNA (ng/L)	455.18 ± 73.64	515.48 ± 48.51	<0.001

Data are presented as means (standard deviation) or number of participants in each group (% of total). CM-CSF, granulocyte–macrophage colony-stimulating factor; IL, interleukin; IFN-γ, interferon-gamma; TNF-α, tumor necrosis factor-alpha; NETs, neutrophil extracellular traps; citH3, citrullinated histone H3; NE-DNA, neutrophil elastase-associated DNA; MPO-DNA, myeloperoxidase-associated DNA. An Independent-samples t-test was used for the current analyses.

In addition, 13 inflammatory indices were measured in the serum of all participants. As shown in Table 2, the non-survivor group showed significantly higher serum levels of granulocyte–macrophage colony-stimulating factor (GM-CSF), interleukin (IL)-1β, IL-2, IL-4, IL-6, IL-8, and IL-10, and tumor necrosis factor-alpha (TNF-*α*) than the survivor group (Table 2, all *p* < 0.05). However, other inflammatory indices showed no significant difference between the two groups.

### Associations of plasma NETs with serum inflammatory indices in SAKI

**Figure 1:**
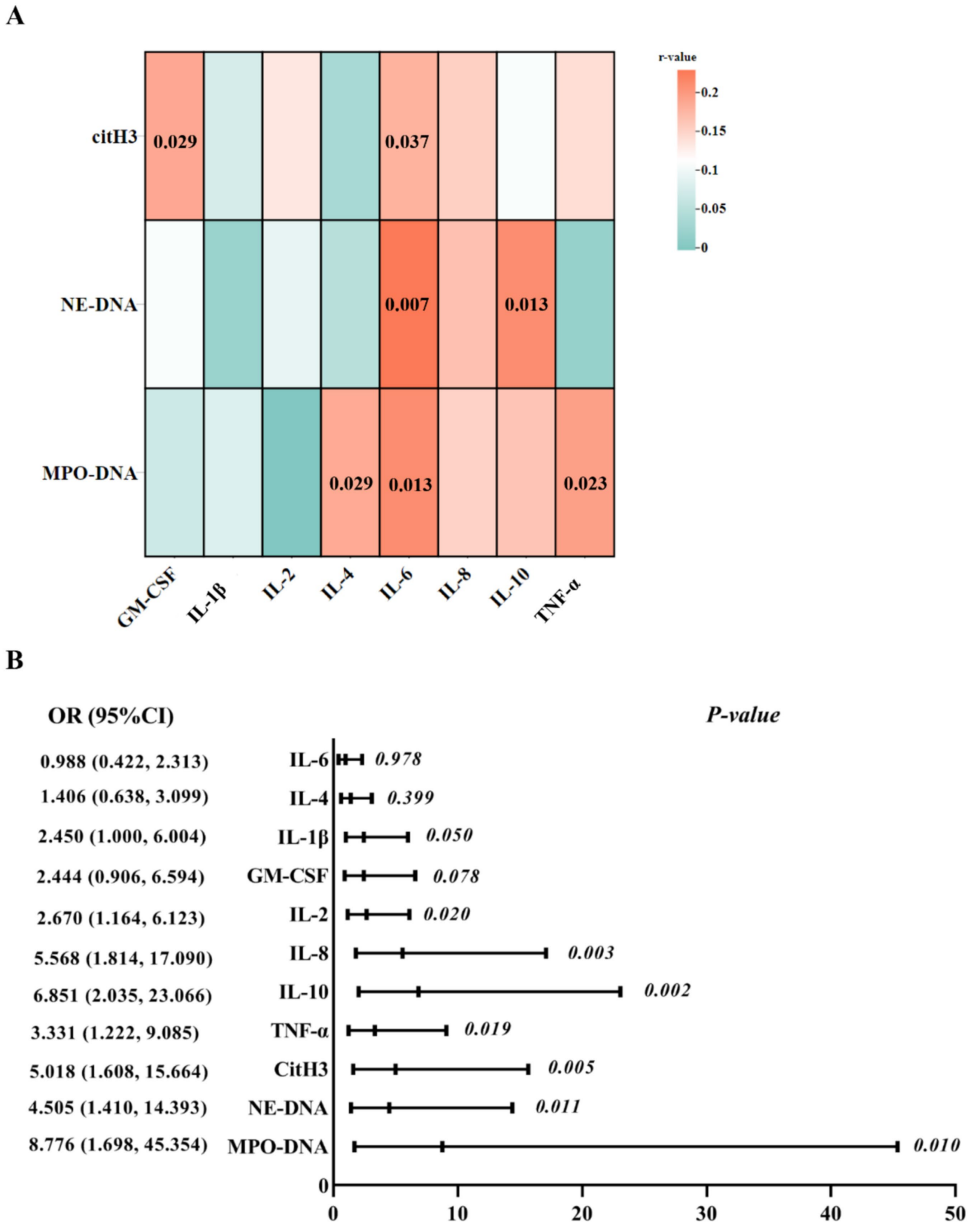
Association analyses of peripheral markers in SAKI patients. (A) The correlation analyses between plasma NET markers and inflammation indicators. The change of color in the square represents the r-values. *p*-values of correlation analyses are marked in the squares. (B) Eight inflammatory indices and three NET markers were included in the logistic regression analysis. SAKI, sepsis-induced acute kidney injury; NETs, neutrophil extracellular traps; IL, interleukin; TNF-*α*, tumor necrosis factor-alpha; CM-CSF, granulocyte–macrophage colony-stimulating factor.

As shown in Figure 1A, there were some significant correlations between plasma NET markers and serum inflammatory indices in SAKI patients. Specifically, the serum IL-6 levels showed a significantly positive correlation with plasma citH3, NE-DNA, and MPO-DNA levels. Meanwhile, the plasma citH3 levels were positively correlated with the serum GM-CSF levels, and the plasma NE-DNA levels showed a positive correlation with the serum IL-10 levels. Furthermore, positive correlations were also found between the plasma MPO-DNA levels and the serum IL-4 and TNF-*α* levels. Additionally, there was a significant correlation of the plasma citH3 levels with the sCr levels (*r* = 0.171, *p* = 0.047); however, the sCr levels were not correlated with the plasma levels of NE-DNA (*r* = 0.166, *p* = 0.053) and MPO-DNA (*r* = 0.089, *p* = 0.302).

Eight differential inflammatory indices and three NET markers were further included in the logistic regression analysis. Multivariate analysis indicated that IL-2, IL-8, IL-10, TNF-*α*, citH3, NE-DNA, and MPO-DNA displayed significant interactive effects on the survival of SAKI patients (Figure 1B).

### ROC curves analysis

**Figure 2:**
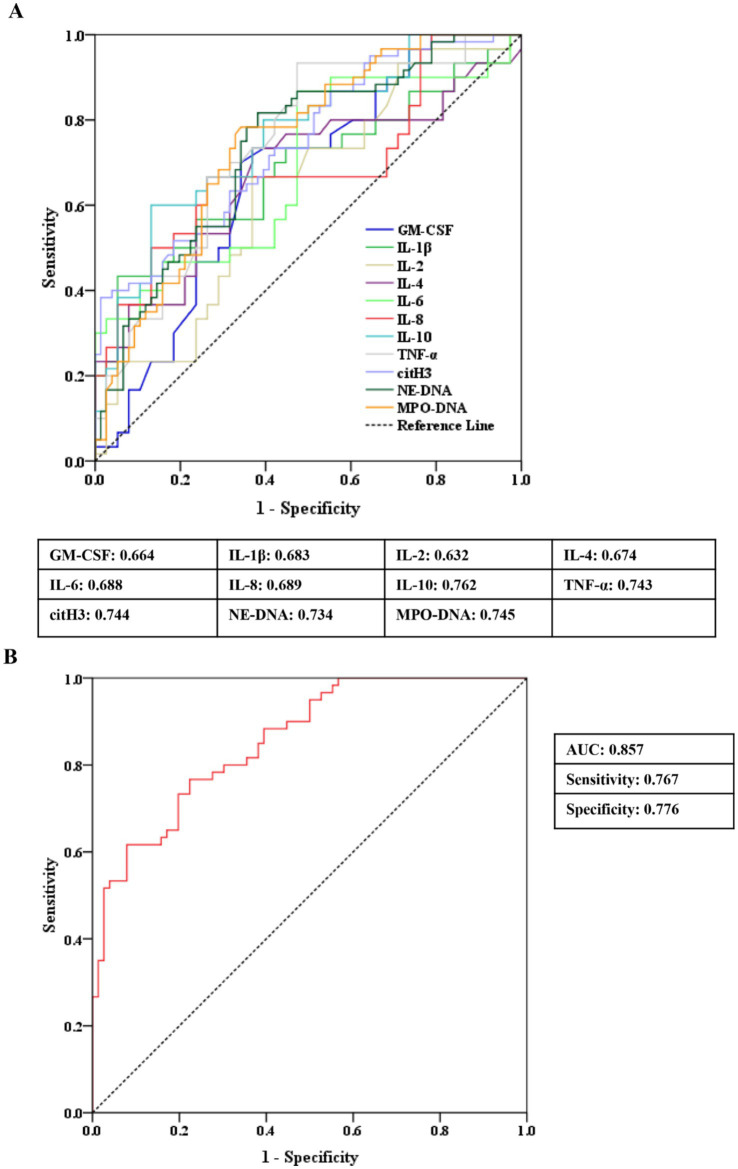
ROC curve analyses of peripheral markers for identifying SAKI non-survivors from SAKI survivors. (A) Eight differential inflammatory indices and three NET markers. (B) Combination of three plasma NET markers. SAKI, sepsis-induced acute kidney injury; NETs, neutrophil extracellular traps; IL, interleukin; TNF-α, tumor necrosis factor-alpha; CM-CSF, granulocyte–macrophage colony-stimulating factor; ROC, receiver operating characteristic; AUC, area under the curve; CI, confidence interval.

Figure 2 displays the area under the curve (AUC) values of eight differential inflammatory indices and three NET markers for distinguishing SAKI patients in the non-survivor group from individuals in the survivor group. Five indicators (i.e., IL-10, TNF-α, citH3, NE-DNA, and MPO-DNA) provided greater diagnostic power, with AUC values >0.70 (Figure 2A; Supplementary Table S1).

To assess the clinical value of plasma NETs for the survival of SAKI, the composite marker of plasma citH3, NE-DNA, and MPO-DNA (citH3 coefficient: 0.035; NE-DNA coefficient: 0.032; MPO-DNA coefficient: 0.017) showed higher AUC values for identifying non-survivors from survivors in SAKI patients (AUC = 0.857) than any single indicators, corresponding to a specificity of 0.776 and a sensitivity of 0.767 (Figure 2B).

### Prognostic analysis for the survival outcomes of sepsis patients

According to the follow-up record, all SAKI patients were evaluated in the present prognostic analysis. Based on potential risk indicators in Figure 2B and basic clinical characteristics and laboratory examinations, the Cox regression model was used to assess baseline factors to predict the 28-day survival outcomes of patients (Table 3). The results indicated that NET markers (citH3, NE-DNA, and MPO-DNA) in plasma may independently predict the survival of SAKI patients (all *p* < 0.001).

**Table 3:** Cox regression for survival analysis.

	HR	95% CI	*P*-value
Sex, male	0.964	0.387–2.401	0.938
Age	0.996	0.975–1.017	0.690
BMI	1.063	0.979–1.154	0.148
Infection sites (pulmonary)*	0.975	0.331–2.865	0.963
Infection sites (urinary tract)*	0.500	0.196–1.275	0.147
Type 2 diabetes (Yes)	0.575	0.270–1.225	0.152
Hypertension (Yes)	0.914	0.437–1.911	0.811
Coronary heart disease (Yes)	1.091	0.585–2.033	0.784
sCr	0.991	0.976–1.006	0.256
eGFR	1.010	0.943–1.083	0.772
BUN	1.003	0.965–1.043	0.866
IL-2	1.360	0.785–2.356	0.273
IL-8	1.007	0.994–1.021	0.282
IL-10	1.141	0.984–1.323	0.082
TNF-α	2.176	0.715–6.620	0.171
citH3	1.021	1.008–1.034	0.001
NE-DNA	1.025	1.009–1.040	0.002
MPO-DNA	1.009	1.003–1.015	0.002

BMI, body mass index; sCr, serum creatinine; BUN, blood urea nitrogen; eGFR, estimated glomerular filtration rate; IL, interleukin; IFN-γ, interferon-gamma; TNF-α, tumor necrosis factor-alpha; NETs, neutrophil extracellular traps; citH3, citrullinated histone H3; NE-DNA, neutrophil elastase-associated DNA; MPO-DNA, myeloperoxidase-associated DNA. *The present variable is a tripartite variable.

**Figure 3:**
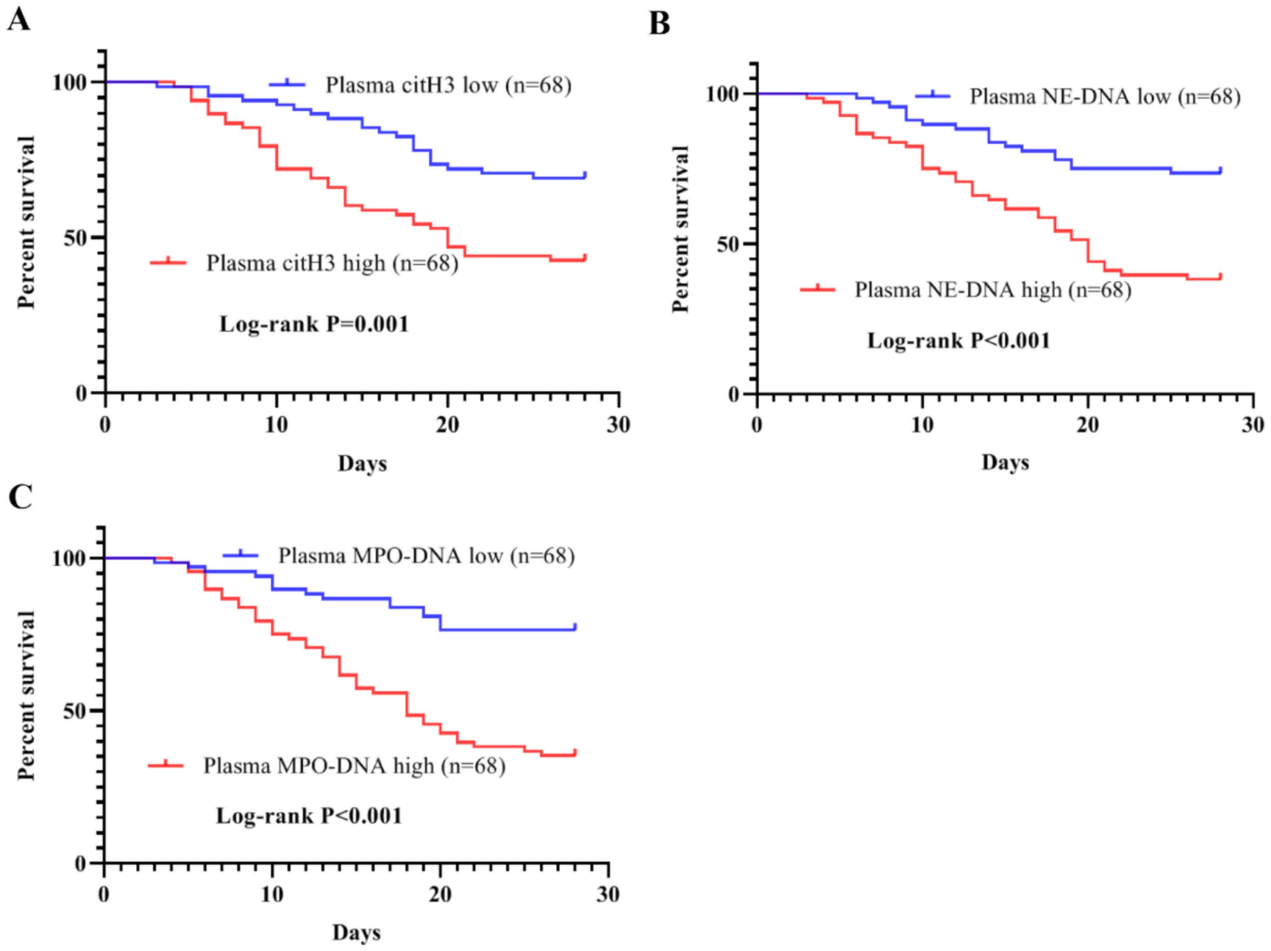
Kaplan–Meier survival curves for SAKI patients with different plasma levels of NETs. (A) Patients with high plasma citH3 levels had a poor 28-day survival (log-rank *p* = 0.001). (B) High plasma NE-DNA levels predicted a poor 28-day survival in SAKI patients (log-rank *p* < 0.001). (C) High plasma MPO-DNA levels were associated with shorter 28-day survival time in patients with SAKI (log–rank *p* < 0.001). SAKI, sepsis-induced acute kidney injury; NETs, neutrophil extracellular traps; citH3, citrullinated histone H3; NE-DNA, neutrophil elastase-associated DNA; MPO-DNA, myeloperoxidase-associated DNA.

To facilitate the survival analysis, plasma citH3, NE-DNA, and MPO-DNA levels were divided into low expression and high expression based on the median values. The Kaplan–Meier survival curves further revealed that patients with higher plasma citH3, NE-DNA, and MPO-DNA levels had significantly poorer 28-week survival than those with lower plasma NET levels (citH3: log-rank *p* = 0.001; NE-DNA: log-rank *p* < 0.001; MPO-DNA: log-rank *p* < 0.001; Figure 3).

## Discussion

To our knowledge, this study represents the first attempt to evaluate the clinical significance of plasma NET levels in SAKI patients. The main findings in our study were as follows: (1) three plasma NET markers showed significantly higher levels in non-survivors as compared to survivors in SAKI patients; (2) in SAKI patients, plasma NET markers were significantly associated with several serum inflammatory indices (i.e., GM-CSF, IL-2, IL-4, and TNF-*α*); (3) IL-2, IL-8, IL-10, TNF-α, citH3, NE-DNA, and MPO-DNA showed an interactive effect on the survival of SAKI patients; (4) plasma NET markers may independently predict the 28-day survival of SAKI patients. Taken together, plasma NET markers may play a role in mediating the inflammatory response, thereby influencing the prognosis of patients with SAKI.

This study identified significant changes in the plasma NET levels between non-survivors and survivors of SAKI. Our findings suggested a correlation between peripheral blood NETs and the severity of SAKI. Compared to other forms of AKI (20, 21), NETs exhibited significant potential as a predictor for survival in cases of sepsis-associated AKI (SAKI). Furthermore, given that both sepsis and AKI independently influence the expression of NETs, SAKI may exert a combined effect on the expression of NETs, thereby enhancing the clinical utility of NETs in SAKI. To determine the underlying mechanism of plasma NETs in the development of SAKI, we further investigated the inflammatory pathway in SAKI. Consistent with some previous studies, SAKI non-survivors had significantly higher serum levels of inflammation indices than survivors, such as IL-1β, IL-6, IL-8, IL-10, and TNF-*α* (32–34). Meanwhile, in the present study, significantly elevated serum GM-CSF, IL-2, and IL-4 levels were first observed in SAKI non-survivors than in the survivors. The present findings showed that there were significant correlations between plasma levels of NETs and serum levels of inflammation factors, which suggested that NETs might modulate acute inflammation in the progress of SAKI (35). Furthermore, the results of multivariate analysis further supported the potential effect between NETs and inflammation on the outcome of SAKI patients. The high serum levels of IL-8 in SAKI can activate and promote neutrophil and monocyte migration toward the area of inflammation (36, 37), and IL-10, as a potent anti-inflammatory cytokine, has a strong immunosuppressive effect on monocytes/macrophages and neutrophils in SAKI (38). Moreover, IL-2 can promote the activation and proliferation of regulatory T cells and B lymphocytes and mediate cellular immunity (39), and GM-CSF can act in a concentration-dependent manner on resident macrophages and migrated monocytes and neutrophils to modify their differentiation/polarization (40). Based on both univariate and multivariate analyses, a synergistic effect between NETs and the inflammatory response was identified as influencing the survival outcomes in SAKI. Therefore, elevated plasma NET levels may exacerbate the inflammatory response, disrupting the balance between pro-inflammatory and anti-inflammatory events and ultimately influencing the prognosis of SAKI.

In our study, three NET markers and two inflammation indices demonstrated AUC values exceeding 0.7 in distinguishing between survivors and non-survivors of sepsis-associated AKI (SAKI). As no single biomarker achieved an accuracy rate above 80% in predicting the outcomes of SAKI patients, we hypothesize that combining the three NET markers may yield superior results. Our findings revealed that a composite marker comprising three plasma NET markers exhibited excellent performance in distinguishing between survivors and non-survivors of SAKI, achieving an AUC value of 0.857. A combined marker may more accurately reflect the true significance of NETs, demonstrating greater potential than any individual inflammatory factor in the clinical management of SAKI.

To further evaluate the clinical significance of the plasma NET marker in predicting 28-day survival outcomes for SAKI patients, a survival analysis was conducted. The results indicated that the plasma NET marker, as a crucial risk factor, could predict the 28-day clinical outcome for SAKI patients. These findings suggested that elevated levels of plasma NET markers in the early stages of SAKI were associated with poorer survival outcomes. Hence, these markers facilitate the identification of SAKI patients who may be at risk of ineffective treatment, acting as an early warning signal.

Our study has several limitations. (1) Plasma NET markers were measured at only one time point. While early evaluations are valuable for predicting treatment effects in SAKI, they do not provide insights into dynamic changes in these markers over time. (2) Although there are direct associations between NET markers and inflammatory indices in SAKI, we cannot solely attribute the severity of the disease to a specific inflammatory reaction. Additional clinical information is essential for predicting clinical outcomes and adjusting treatment strategies in SAKI in a timely manner. (3) The present study was conducted at a single center. A multicenter study is necessary to further validate our findings.

## Conclusion

The present study demonstrated that plasma NET markers in the early stage of SAKI could predict the survival outcome of SAKI patients, which may be involved in the inflammatory response. Meanwhile, higher plasma levels of NET markers were strongly associated with shorter survival time in SAKI patients. The current findings may provide a novel non-invasive biomarker for the prognosis of SAKI, as well as novel ideas for further research about the pathogenesis of SAKI.

## Data Availability

The original contributions presented in the study are included in the article/Supplementary material, further inquiries can be directed to the corresponding authors.

## Footnotes

^1^ https://sample-size.net/
