# Supplementary material for: Plasma neutrophil extracellular traps in patients with sepsis-induced acute kidney injury serve as a new biomarker to predict 28-day survival outcomes of disease
Source: Front Med (Lausanne). 2024 Nov 19;11:1496966. doi: 10.3389/fmed.2024.1496966 (PMC11611547; doi:10.3389/fmed.2024.1496966)
Supplement: Supplementary file 1 [file Data_Sheet_1.docx]

**Sample Size Calculation**

The sample size calculation was performed using an online sample size calculators (<https://sample-size.net/>). We performed the sample size calculation (α=0.05, β=0.2) to confirm whether 76 SAKI survivors and 60 SAKI non-survivors was acceptable based on the detected results of significant changed three NETs and eight inflammation factors. The results of sample size calculation are as following:

citH3: N_total_ = 36, N_group 1_ = 18, N_group 2_ = 18, Power = 0.803;

MPO-DNA: N_total_ = 36, N_group 1_ = 18, N_group 2_ = 18, Power = 0.805;

NE-DNA: N_total_ = 40, N_group 1_ = 20, N_group 2_ = 20, Power = 0.812;

GM-CSF: N_total_ = 86, N_group 1_ = 43, N_group 2_ = 43, Power = 0.802;

IL-1β: N_total_ = 74, N_group 1_ = 37, N_group 2_ = 37, Power = 0.810;

IL-2: N_total_ = 120, N_group 1_ = 60, N_group 2_ = 60, Power = 0.806;

IL-4: N_total_ = 98, N_group 1_ = 49, N_group 2_ = 49, Power = 0.807

IL-6: N_total_ = 68, N_group 1_ = 34, N_group 2_ = 34, Power = 0.808

IL-8: N_total_ = 52, N_group 1_ = 26, N_group 2_ = 26, Power = 0.812

IL-10: N_total_ = 36, N_group 1_ = 18, N_group 2_ = 18, Power = 0.812

TNF-α: N_total_ = 38, N_group 1_ = 19, N_group 2_ = 19, Power = 0.821

Therefore, the present subjects including 76 SAKI survivors and 60 SAKI non-survivors can provide an acceptable power (> 80%), and the sample size using in the present study can meet the minimum sample requirement based on results of the sample size calculation.

**Detailed explanation of excluded standard partial diseases**

Severe cardiovascular and cerebrovascular diseases refer to patients with end-stage organic heart disease and stroke patients with a GCS score of 3. Severe malnutrition refers to a PG-SGA score of ≥9 (Grade C). Severe trauma refers to trauma patients with an ISS score of ≥25. Severe immunodeficiency refers to a CD4 lymphocyte count of <200 cells/μl. Severe hepatitis refers to diffuse necrosis of liver tissue caused by hepatitis virus infection, leading to acute liver failure, confirmed by serological or virological tests, and meeting all of the following criteria: (1) severe jaundice or rapidly worsening jaundice; (2) hepatic encephalopathy; (3) B-mode ultrasound or other imaging studies showing rapid shrinkage of liver volume; (4) progressive deterioration of liver function indicators.

**Supplementary Table 1. ROC curves analyses in SAKI patients**

|  | AUC (95% CI) | Sensitivity (%) | Specificity (%) |
| --- | --- | --- | --- |
| GS-CSF | 0.664 (0.579 - 0.743) | 70.00 | 65.75 |
| IL-1β | 0.683 (0.598 - 0.760) | 43.33 | 94.74 |
| IL-2 | 0.632 (0.545 - 0.713) | 66.67 | 63.16 |
| IL-4 | 0.674 (0.589 - 0.752) | 73.33 | 63.16 |
| IL-6 | 0.688 (0.603 - 0.764) | 86.67 | 52.63 |
| IL-8 | 0.689 (0.605 - 0.766) | 66.67 | 73.68 |
| IL-10 | 0.762 (0.684 - 0.833) | 60.00 | 86.84 |
| TNF-α | 0.743 (0.661 - 0.814) | 93.33 | 52.63 |
| citH3 | 0.744 (0.662 - 0.815) | 38.33 | 98.68 |
| NE-DNA | 0.734 (0.652 - 0.806) | 81.67 | 61.84 |
| MPO-DNA | 0.745 (0.663 - 0.816) | 78.33 | 65.79 |

SAKI, sepsis-induced acute kidney injury; NETs, neutrophil extracellular traps; IL, interleukin; TNF-α, tumour necrosis factor alpha; CM-CSF, granulocyte-macrophage colony-stimulating factor; ROC, receiver operating characteristic; AUC, area under the curve; CI, confidence interval.


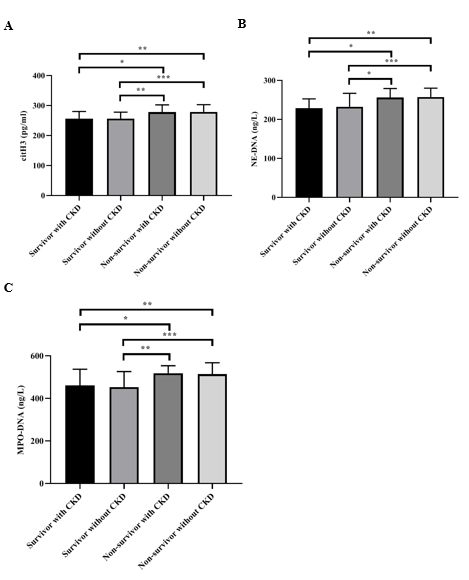


**Supplementary Figure 1. Comparison of three plasma NETs levels among SAKI patients with and without CKD**
